# Editorial: Women in radiology: neuroimaging and neurotechnology

**DOI:** 10.3389/fradi.2025.1643898

**Published:** 2025-06-30

**Authors:** Laura Elin Pigott, Katya Mileva, Laura Mancini

**Affiliations:** ^1^ College of Health and Life Sciences, London South Bank University, London, United Kingdom; ^2^ Department of Translational Neuroscience and Stroke, Queen Square Institute of Neurology, University College London, London, United Kingdom; ^3^ TU London, Teesside University London, London, United Kingdom; ^4^ Lysholm Department of Neuroradiology, National Hospital for Neurology and Neurosurgery, University College London Hospitals NHS Foundation Trust, London, United Kingdom

**Keywords:** neuroscience, radiology, artificial intelligence, neuroimaging, imaging, brain science, women, equality

**Editorial on the Research Topic**
Women in radiology: neuroimaging and neurotechnology

## Introduction

Despite notable progress, women remain underrepresented across STEM disciplines, including neuroscience and radiology. Globally, women constitute about one-third of the research workforce (1). In neuroscience, women comprise over half of doctoral candidates, yet they occupy fewer than 14% of tenure-track faculty positions (2). A similar disparity is observed in radiology, where women account for approximately 25% of practicing radiologists and just 13% of leadership roles (3). These disparities underscore the persistent gender gap stunting scientific progress, as without gender equality in science; scientific progress can fulfil just half of its potential (4).

Neuroimaging lies at the nexus of technological innovation, clinical application, and data-driven neuroscience. As each of these modalities becomes increasingly vital for diagnosing, monitoring, and treating neurological conditions, it is imperative that their evolution is guided by scientific rigor, a commitment to inclusivity, and recognition of the diversity of our global community. This Research Topic was therefore devised with a dual objective: to critically examine the principal methodological challenges for the future development and clinical translation of neuroimaging and neurotechnology, while concurrently celebrating the pivotal contributions of women researchers driving innovation in this dynamic and interdisciplinary domain.

## Issues of concern

The neuroimaging sector is a rapidly growing, with the global neuroimaging market projected to grow at a compound annual rate of approximately 6% CAGR between 2024 and 2030, rising from an estimated $37.6 billion in 2023 to $56.6 billion by 2030. This growth is driven by technological advancements and the increasing demand for brain diagnostics, particularly as the population ages (5). However, the field faces substantial methodological and infrastructural challenges that must be addressed to ensure sustained progress.

Leading amongst these challenges is the lack of standardisation in neuroimaging methods and data practices; an issue widely recognised as part of the need for innovation in the PET/MR technology and applications at this year's meeting of The International Society for Magnetic Resonance in Medicine (6). The absence of harmonised protocols undermines reproducibility, compromises diagnostic accuracy, and impedes cross-institutional collaborations. Standardised data formats and acquisition protocols are essential to ensure that findings can be reliably replicated across different research settings and imaging scanners. Without standardisation, even rigorously conducted studies may yield results that are hard to compare or generalise, thereby limiting their scientific utility.

Moreover, consistent imaging practices enhance diagnostic precision by minimising methodological variability and ensuring that observed differences reflect true biological variation rather than technical artefacts. As demonstrated in the context of brain disorders, a standardised diagnostic approach can expand access to high-quality diagnostic services and reduce subjective interpretation errors (7), ultimately improving patient outcomes. Moreover, standardisation facilitates collaboration by lowering technical and financial barriers for investigators in resource-limited settings. When data and tools are shared in interoperable formats, multi-centre collaborations become more feasible, and large-scale datasets can be aggregated to enable insights that can be generalised across diverse populations. This alignment of neuroimaging practices fosters inclusive collaboration and drives equitable progress (8).

## Key insights from contributions

### Neuroimaging and personalised medicine

By pairing standardised, high-resolution T1-weighted MRI with graph-theoretical Liu et al. explore sex-specific morphological and network-level differences in patients with Parkinson's disease with and without possible REM sleep behaviour disorder. The authors uncover sex-related disparities in grey matter and network integration, emphasising the clinical significance of sex-aware neuroimaging research.

In parallel Deng et al. offer a systematic review of effective connectivity (EC) studies in adolescent depression, underscoring the promise of EC in identifying causal network abnormalities in vulnerable populations. The authors argue for consistency in resting-state fMRI acquisition and EC modelling as a prerequisite for translational insight. Together, these works highlight how standardisation in imaging acquisition and analytical frameworks can enhance diagnostic sensitivity, prognostic value and equity in neuroscience research.

### Predictive modelling and standardisation challenges

Radiomics and machine learning offer powerful, non-invasive tools for neuro-oncological prediction, yet their clinical translation hinges on standardisation. In their systematic review and meta-analysis, Chung and Pigott evaluate machine learning-based models predicting IDH and ATRX mutations in gliomas across 11 studies. Despite reporting high pooled AUC values (0.948 for IDH and 0.842 for ATRX), the authors identify major limitations: inconsistent imaging protocols, heterogeneity in feature extraction, and a lack of reproducibility. Their findings call for harmonising machine learning pipelines and data annotation to unlock the full clinical potential of AI-assisted diagnostics.

Hakhu et al. confront a related issue in their study by comparing electrical conductivity estimates derived from various diffusion MRI models. Using the same datasets, they demonstrate how differing diffusion microstructure models produce divergent conductivity estimates, revealing the substantial interpretive variability introduced by methodological choice.

Both contributions point to a central challenge in modern neuroimaging: without community-driven, international standards for imaging protocols, model validation, and metadata annotation, predictive modelling risks inconsistency and inequity. These studies advocate for open science frameworks where reproducibility and interoperability are the default, not the exception.

### Clinical utility and prognostic power

The role of neuroimaging in acute and subacute neurological disorders is no longer limited to diagnosis; it now holds promise for early prognostication. Abbuehl et al. retrospectively analysed MRI data from 129 cases of viral or idiopathic meningoencephalitis, identifying early imaging markers that correlate with long-term functional outcomes. These findings suggest that prognostic models based on MRI could be developed, but only if acquisition protocols and interpretation thresholds are consistent across centres.

Similarly, the meta-analysis of Zhi et al. compares the diagnostic utility of neuroendoscopy and small-bone-window craniotomy in hypertensive intracerebral haemorrhage. Neuroendoscopy consistently demonstrated superior outcomes, however, the variability in outcome definitions, surgical imaging methods, and follow-up intervals across trials, complicates synthesis and translation.

The principle conclusion from these studies is that image-driven prognostication is feasible but must rest on reproducible, quantifiable endpoints - standardised in both acquisition and interpretation stages. Only then can we develop equitable, evidence-based tools for early risk stratification and intervention planning in neurological care.

### Considering optimal imaging for conditions

Lolli et al. offer a timely review of CT perfusion (CTP) imaging in aneurysmal subarachnoid haemorrhage, examining its utility in detecting delayed cerebral ischaemia and guiding endovascular rescue therapy. Despite CTP's widespread use, the authors identify critical limitations: inconsistent software tools, lack of validated perfusion thresholds, and discrepancies with stroke imaging protocols. These shortcomings illustrate a broader point echoed across this Research Topic; neuroimaging cannot effectively guide clinical intervention unless its outputs are rigorously standardised, validated, and reproducible. This review underscores the need for consensus-building and harmonisation in radiology, ensuring patient care is informed by consistent and comparable data.

## Conclusion

The studies featured in this Research Topic showcase how research led by women scientists' aids in pushing neuroimaging and neurotechnology advancement forward with more rigorous, standardised practices. The articles exemplify their outstanding scientific excellence and leadership, as they tackle major contemporary challenges in neuroimaging and neurotechnology. These challenges range from converging machine learning pipelines across institutions to refining clinical imaging criteria for diagnosis and treatment. By confronting issues such as reproducibility in AI algorithms and consistency in clinical interpretations, the women-led teams in this issue are setting new benchmarks for research. Their contributions underscore that improving technical and clinical standards is integral to advancing neuroimaging and neurotechnology. When women and other underrepresented researchers are also at the forefront of science, they bring fresh perspectives that spur novel approaches to longstanding problem.
